# Intra-peer violence, socioeconomic and behavioral characteristics of sexual and gender diverse adolescents: a cross-sectional study^*^

**DOI:** 10.1590/1518-8345.7920.4864

**Published:** 2026-07-20

**Authors:** Micael Franco Alves, Franciele Marabotti Costa Leite

**Affiliations:** 1 Universidade Federal do Espírito Santo, Vitória, ES, Brazil.

**Keywords:** Adolescents, Gender Diversity, Bullying, Cyberbullying, Violence, Socioeconomic Profile

## Abstract

**Objective:**

to identify the socioeconomic and behavioral profile of adolescents who self-identify as lesbian, gay, bisexual, transvestite, transgender, and others, as well as their experiences of peer violence and its motivations.

**Method:**

a cross-sectional, descriptive study was conducted among 949 adolescents aged 14 to 19 years, using self-administered electronic questionnaires, which were analyzed using crude and relative frequencies and 95% confidence intervals.

**Results:**

cisgender adolescents (81.0%), middle-class students (44.7%), Black or Brown students (57.4%), and public-school students (67.2%) predominated. The prevalence of bullying was 67.8%, with 13.0% motivated by sexual orientation, and cyberbullying was identified in 11.5%. Condom use was higher during the first sexual intercourse (50.7%) than during the last (40.3%). Current alcohol use was around 47.2%, cigarette use was 9.1%, and drug use was 15.1%.

**Conclusion:**

it is concluded that the adolescents in this study face high rates of violence, social exclusion, and behavioral vulnerabilities, reinforcing the need for integrated strategies to promote inclusive and safe environments, as well as public policies aimed at equity and health for this population.

## Highlights

**(1)** It was found that 67.8% of LGBT+ adolescents reported being victims of bullying. **(2)** Thirteen percent of individuals reported suffering bullying motivated by sexual orientation. **(3)** Condom use was higher in the first sexual encounter (50.7%) than in the last (40.3%). **(4)** A total of 47.2% of participants reported current alcohol consumption. **(5)** Data reinforce the need for policies for safe and inclusive environments

## Introduction

Adolescence is a crucial period of development, characterized by significant physical, psychological, and social changes. At this stage, identity formation and the search for balance in the face of change play essential roles in the development of an individual’s intellect, personality, and social skills^1^. Sexual orientation, which is shaped by a combination of biological, psychological, social, cultural, and family factors, emerges as a central issue during this phase. Understanding this construction is fundamental to providing adequate support to adolescents as they explore and define their identities^2^.

However, in a social context often marked by heteronormative standards, lesbian, gay, bisexual, transgender, queer, intersex, asexual, pansexual, and non-binary (LGBTQIAPN+) adolescents face unique challenges, such as family rejection, bullying, and discrimination. The acronym LGBTQIAPN+* encompasses a wide diversity of gender identities and sexual orientations, including lesbians (women who have relationships with women), gays (men who have relationships with men), bisexuals (attraction to more than one gender), transvestites and transsexuals (whose gender identity differs from the sex assigned at birth) , queer (identity that deviates from the heteronormative norm), intersex (people with variations in sexual characteristics), asexual (individuals who do not feel sexual attraction), pansexual (attraction regardless of gender), and non-binary (people who do not identify exclusively as male or female)^2^. Such experiences can compromise these individuals’ psychological well-being and hinder the formation of a secure and positive identity^3^. In this scenario, it is essential to understand the social and psychological dynamics among these adolescents to promote supportive, inclusive, and protective environments.

Violence, in its various forms-physical, sexual, and psychological-is a critical public health issue during adolescence. Its prevalence is amplified by risk factors such as family dynamics, socioeconomic status, and adverse social environments. These factors must be considered when developing effective prevention strategies^4^. In particular, bullying directed at LGBT+ adolescents has been shown to be a growing concern. Studies indicate that these young people experience more severe victimization, with multiple trajectories of aggression that directly impact their mental health and well-being^5^.

The vulnerability of adolescents is intensified by factors such as gender identity, sexual orientation, and race, creating additional disparities in exposure to violence, especially for those with multiple marginalized identities^6^.

In this context, health and education professionals play an essential role in supporting LGBT+ adolescents. Through educational initiatives, awareness campaigns, and the development of safe and inclusive spaces, these professionals can help mitigate the impacts of discrimination and promote mental health and well-being. Integrated strategies between the health and education sectors are fundamental to preventing violence and building a more equitable society^7^.

Given this scenario, the objective of this article is to identify the socioeconomic and behavioral profile of adolescents who self-identify as lesbian, gay, bisexual, transvestite, transgender, and others, as well as their experiences of peer violence and its motivations.

## Method

### Study design

This is a descriptive cross-sectional study conducted in 63 public and private high schools located in the Metropolitan Region of Greater Vitória (RMGV, acronym in Portuguese), Espírito Santo, covering the municipalities of Cariacica, Fundão, Guarapari, Serra, Viana, Vila Velha, and Vitória.

### Location

The study was conducted in 38 public and 25 private schools in the RMGV, Espírito Santo, Brazil, which were randomly selected, for a total of 63 schools. Currently, this region has 171 public and/or private high schools; thus, the schools in which we conducted the study comprise 36.8% of the total.

### Period

Data collection took place between March and December 2023.

### Population

The total population interviewed was 4,614 adolescents enrolled in high school, with a sample of 949 students who self-identified as LGBT+ for the present study. Cognitive deficits that prevented understanding of the questionnaire were adopted as exclusion criteria. For the present study, adolescents are defined as individuals aged 10-19, according to the World Health Organization^8^.

### Sample definition

This study is an excerpt from a larger study entitled State Survey of School Health in Espírito Santo (PESC, acronym in Portuguese), in which the sample, designed to ensure comparative representativeness between the public and private networks, employed stratification by network type. Simple samples were then obtained for each stratum and subsequently converted to complex samples using SPSS 26, with a 95% confidence level and a maximum error of 5%. In addition, for each municipal stratum, parameters of 95% confidence and a maximum error of 10% were established. Sampling was performed using a cluster design with stratification into three strata, with a 95% confidence level and a maximum allowable error of 5%. For each municipal stratum, the maximum error was 10%.

### Study variables

The variables analyzed included: socioeconomic variables: age (14/15, 16/17, 18/19 years); gender identity, in which cisgender was classified as gender identity corresponding to that assigned at birth, classified as cisgender or non-cisgender; race/skin color (white, black, yellow, brown, indigenous); in a relationship (no, yes); type of school (public, private); socioeconomic classification (A, B, C, D/E) - for this variable, we used the criteria of the Brazilian Association of Research Companies^9^; religion (none, Catholic, Evangelical, Spiritist, African roots, other); has a job/internship (no, yes); parents’ status (living together, living apart, one of them has died - or both); behavioral: has had sexual intercourse (no, yes); condom use during first intercourse (no, yes); condom use during last sexual intercourse (no, yes); drug experimentation (no, yes); current drug use (no, yes); cigarette experimentation (no, yes); current cigarette use (no, yes); alcohol experimentation (no, yes); current alcohol use (no, yes); peer violence: victim of bullying (no, yes); practiced bullying (no, yes); victim of cyberbullying (no, yes); practiced cyberbullying (no, yes); frequency of involvement in physical fights (never involved, involved once or more); types of bullying suffered: hit/bumped/kicked/locked in closed places/pushed (no, yes); teased because of race, nationality, or skin color (no, yes); was teased because of religion (no, yes); was teased through jokes or comments of a sexual nature or obscene gestures (no, yes); was deliberately excluded from activities or ignored completely (no, yes); was teased because of the appearance of their body or face (no, yes); suffered another form of bullying (no, yes); motivation for bullying: skin color/race (no, yes); religion (no, yes); facial appearance (no, yes); physical appearance (no, yes); sexual orientation (no, yes); region of origin (no, yes); other (no, yes).

### Instruments used to collect information

Data were collected using self-completed electronic questionnaires on tablets via the REDCap (Research Electronic Data Capture) system, which included blocks for socioeconomic and behavioral characterization. In addition, experiences of peer violence and their motivations were tracked using an instrument based on the Adverse Childhood Experiences International Questionnaire (ACE-IQ), adapted and validated for use in Brazil^10^.

### Data collection

Data collection was conducted by a team responsible for ensuring the quality and integrity of the information, which was trained and supervised. Before the start of the research, authorization was requested and obtained from the State Department of Education (SEDU) to conduct the study in the selected public schools. Subsequently, prior contact was established with public institutions, as well as private schools, to present the proposal at a seminar held in March 2023, which was attended by approximately 80% of the schools selected for the research. On this occasion, the objectives and procedures of the research were presented, and the Free and Informed Consent Forms (FICF) were delivered to the representatives of the institutions. For the absent schools, the team made in-person visits, ensuring 100% adherence of the selected units. Afterward, a new contact was established to collect the FICF from the schools and to schedule data collection, which took place in classrooms and/or auditoriums, depending on the school’s availability. It is important to note that, at the time of data collection, the team distributed a tablet to each participating student and provided guidance on its use. The average time to complete the questionnaire was about fifty minutes.

### Data processing and analysis

Descriptive statistical analyses were conducted using crude frequency, relative frequency, and 95% confidence intervals (95% CI). All analyses were performed using Stata 17.0.

### Ethical aspects

This study was approved by the Research Ethics Committee of the Federal University of Espírito Santo, in accordance with Resolution 466/2012 of the National Health Council. Parents and/or guardians of participating adolescents signed the Free and Informed Consent Form (FICF), and adolescents signed the Free and Informed Assent Form (AF). It should be noted that this research was funded by the Espírito Santo Research Support Foundation (FAPES, in Portuguese) under case number CAAE 65545622.5.0000.5060.

## Results

The socioeconomic characteristics of self-identified LGBT+ students in the RMGV (Table 1) indicated that the majority identified as Black (Brown - 38.9%; Black - 18.5%). The majority were adolescents aged 16-17 (65.5%), not in a relationship (69.0%), cisgender (81.0%), and attending public schools (67.2%).

It should be noted that 44.7% belonged to class B and 45.3% were nonreligious. Finally, 76.7% were unemployed, and among their parents, 47.1% were separated.

**Table 1:** Socioeconomic characteristics of self-declared LGBT+ schoolchildren. Vitória, ES, Brazil, 2023

Variable	N*	%^†^	95% CI^‡^
Age group (years)			
14 to 15	204	21.5	19.0-24.2
16 to 17	621	65.5	62.4-68.5
18 to 19	123	13.0	11.0-15.2
Gender identity			
Cisgender	769	81.0	78.4-83.4
Non-cisgender	180	19.0	16.6-21.6
Race/Color			
White	380	40.0	37.0-43.2
Black	175	18.5	16.1-21.0
Yellow	15	1.6	0.9-2.0
Brown	369	38.9	35.8-42.0
Indigenous	10	1.0	0.5-2.0
Are you in a relationship?			
No	655	69.0	66.0-71.8
Yes	294	31.0	28.1-33.9
Public or private school?			
Public	638	67.2	64.1-70.1
Private	311	32.8	29.9-34.6
Socioeconomic classification			
A	154	16.3	14.0-18.7
B	424	44.7	41.6-47.9
C	317	33.5	30.5-36.5
D/E	53	5.5	4.2-7.2
Religion			
None	429	45.3	42.0-48.4
Catholic	167	17.6	15.3-20.1
Evangelical	215	22.6	20.1-25.4
Spiritist	54	5.7	4.4-7.4
African Roots	48	5.0	3.8-6.6
Other	36	3.8	2.7-5.2
Has a job/internship			
No	728	76.7	74.0-79.3
Yes	221	23.3	20.7-26.0
Parental status			
Living together	424	45.1	41.9-48.3
Living apart	443	47.1	43.9-50.3
One or both parents deceased	73	7.8	6.2-9.6

*N = Absolute frequency; ^†^% = Prevalence; ^‡^95% CI = 95% Confidence interval

The behavioral characteristics of self-declared LGBT+ students in the RMGV are presented in Table 2. Regarding sexual experience, the majority (56.6%) had never had sexual intercourse, while 43.4% reported having had sex. Condom use during first sexual intercourse was reported by approximately 51.0% of adolescents (95% CI: 45.9-55.5); however, during last sexual intercourse, approximately 40.0% reported using condoms (95% CI: 44.4-54.1). Regarding lifetime drug use, 33.6% had used drugs, and 15.1% currently use them. It was found that 31.8% had smoked, and currently, this frequency is 9.1%. Regarding lifetime alcohol use, 77.2% had consumed alcohol, and 47.2% currently use it.

**Table 2:** Behavioral characteristics of self-declared LGBT+ schoolchildren. Vitória, ES, Brazil, 2023

Variable	N*	%^†^	95% CI^‡^
Have you ever had sexual intercourse?			
No	537	56.6	53.4-59.7
Yes	412	43.4	40.3-46.6
Use of condoms during first sexual intercourse			
No	203	49.3	44.4-54.1
Yes	209	50.7	45.9-55.5
Use of a condom during last sexual intercourse			
No	246	59.7	54.9-64.3
Yes	166	40.3	35.7-45.1
Drug experimentation			
No	630	66.4	63.3-69.3
Yes	319	33.6	30.7-36.7
Current drug use			
No	805	84.8	82.4-87.0
Yes	144	15.2	13.0-17.6
Cigarette experimentation			
No	647	68.2	65.1-71.0
Yes	302	31.8	29.0-34.8
Current cigarette use			
No	863	90.9	89.0-92.6
Yes	86	9.1	7.3-11.0
Alcoholic beverage tasting			
No	216	22.8	20.2-25.6
Yes	731	77.2	74.4-79.7
Current alcohol consumption			
No	500	52.8	49.6-56.0
Yes	447	47.2	44.0-50.4

*N = Absolute frequency; ^†^% = Prevalence; ^‡^95% CI = 95% confidence interval

Regarding the prevalence of peer violence (Table 3), 67.8% of LGBT+ students reported having been victims of bullying, and 11.5% reported having experienced cyberbullying. Regarding bullying, 15.2% of participants admitted to having committed acts of this nature, while 7.0% reported engaging in cyberbullying. In addition, about 38.0% reported involvement in physical fights at least once.

**Table 3:** Prevalence of bullying and cyberbullying among schoolchildren who self-identify as LGBT+. Vitória, ES, Brazil, 2023

Variable	N*	%^†^	95% CI^‡^
Victim of bullying			
No	306	32.2	29.3-35.3
Yes	643	67.8	64.7-70.7
Bullied			
No	803	84.8	82.4-86.9
Yes	144	15.2	13.1-17.6
Victim of cyberbullying			
No	838	88.5	86.3-90.4
Yes	109	11.5	9.6-13.7
Practiced cyberbullying			
No	881	93.0	91.2-94.5
Yes	66	7.0	5.5-8.8
The frequency with which you have been involved in physical fights			
Never got involved	569	62.3	59.1-65.3
Was involved one or more times	345	37.7	34.7-40.9

*N = Absolute frequency; ^†^% = Prevalence; ^‡^95% CI = 95% confidence interval

Regarding the types of bullying (Table 4), it was observed that 8.0% of participants reported having suffered physical aggression, such as being kicked, pushed, or locked in closed spaces. Insults related to race, nationality, or skin color were mentioned by approximately 19.0%. Religious comments were reported by 5.5%, and obscene jokes or gestures of a sexual nature were reported by 17.3%. In addition, 29.2% reported being excluded from activities or entirely ignored, while 44.3% suffered insults related to physical appearance, and other forms of bullying were identified by 12.9%.

**Table 4:** Types of bullying practiced against schoolchildren who self-identify as LGBT+. Vitória, ES, Brazil, 2023

Variable	N*	%^†^	95% CI^‡^
Hit/Bumped into/Kicked/Locked in closed spaces/Pushed.			
No	873	92.0	90.0-93.5
Yes	76	8.0	6.4-10.0
They were teased (mocked) because of their race, nationality, or skin color.			
No	770	81.1	78.5-83.5
Yes	179	18.9	16.5-21.5
They mocked him/her because of his/her religion.			
No	897	94.5	92.8-95.8
Yes	52	5.5	4.2-7.1
They were teased (mocked) through jokes or comments of a sexual nature or obscene gestures.			
No	785	82.7	80.1-85.0
Yes	164	17.3	15.0-19.9
Was excluded from activities on purpose or completely ignored			
No	672	70.8	67.8-73.6
Yes	277	29.2	26.4-32.2
They were teased (mocked) because of the appearance of their body or faces.			
No	529	55.7	52.6-58.9
Yes	420	44.3	41.1-47.4
I suffered another form of bullying.			
No	827	87.1	84.8-89.1
Yes	122	12.9	10.9-15.1

*N = Absolute frequency; ^†^% = Prevalence; ^‡^95% CI = 95% confidence interval

The analysis of the motivations for bullying among LGBT+ adolescents (Table 5) revealed that skin color or race was mentioned by 5.0% of participants, while religion was reported by 4.2%. Regarding facial appearance, 9.1% of adolescents reported having been bullied for this reason, and 10.4% indicated body appearance as a motivating factor. It is worth noting that sexual orientation had the highest prevalence among the reasons presented, being cited by 13.0% of participants.

**Table 5:** Motivations for bullying against self-declared LGBT+ students. Vitória, ES, Brazil, 2023

Variable	N*	%^†^	95% CI^‡^
Color/Race			
No	902	95.0	93.4-96.2
Yes	47	5.0	3.7-6.5
Religion			
No	909	95.8	94.3-96.8
Yes	40	4.2	3.1-5.6
Face appearance			
No	863	90.9	88.9-92.6
Yes	86	9.1	7.4-11.0
Body appearance			
No	850	89.6	87.4-91.3
Yes	99	10.4	8.6-12.5
Sexual orientation			
No	826	87.0	84.7-89.0
Yes	123	13.0	10.9-15.2
Region of origin			
No	922	97.1	95.8-98.0
Yes	27	2.8	1.9-4.1
Others			
No	862	90.8	88.8-92.5
Yes	87	9.2	7.4-11.1

*N = Absolute frequency; ^†^% = Prevalence; ^‡^95% CI = 95% confidence interval

## Discussion

The socioeconomic data presented reveal important aspects about the profile of self-declared LGBT+ schoolchildren in Espírito Santo. It is observed that 65.5% were in the age group of 16 and 17 years, predominantly cisgender (81.0%), which reflects the majority trend in many societies, although a significant portion (19.0%) identified themselves as non-cisgender, highlighting the diversity of gender identities within this group^11^. In addition, 69.0% of the participants were not in a relationship, which may indicate a pattern of relationships or a stage of life in which most have not yet been involved in formal relationships, something common in younger age groups^12^.

In terms of religion, 45.3% of adolescents reported being without religion. Many LGBT+ adolescents experience a conflict between their sexual and religious identities, which often results in feelings of disconnection from their spiritual communities^11^. They report experiences of initial connections with their faith, but later begin to question their beliefs due to their sexual orientation^13^.

When comparing the data obtained in the research with other studies in Brazil, it can be observed that the prevalence of sexual initiation in the present study among adolescents in the sample is considerably higher (43.4%). A survey conducted in Piauí, with adolescents without sexual orientation criteria, indicated a prevalence of 24.2%^14^, and in Curitiba, also with the general adolescent population, the prevalence was 22.9%^15^. These data suggest that sexual initiation is a phenomenon present in LGBT+ adolescence, with sexual orientation as a factor that can influence the age of initiation, reinforcing the importance of sex education programs for this population.

In this study, approximately 51.0% of participants reported using condoms during their first sexual intercourse, a finding higher than that identified in a study with adolescents from the general population (24.7%)^16^. On the other hand, during their last sexual intercourse, the prevalence of condom use was 40.3%, a percentage lower than that found in another study conducted with adolescents from the general population (68.8%)^17^. These findings suggest that LGBT+ adolescents tend to adopt greater protection at the beginning of their sexual life, motivated mainly by the prevention of STIs, mistrust, or imposition by their partner. However, the reduction in condom use in subsequent sexual encounters may be related to trust in the partner, personal preferences, forgetfulness, or unavailability of the method^18^.

Considering the above, it is essential to draw attention to the health education work that needs to be carried out in schools by health professionals, precisely so that adherence to contraceptive methods is extended beyond the first sexual intercourse, since this action improves adolescents’ understanding of sexual health, leading to good attitudes and behaviors in relation to STIs^19^.

As for drugs, 33.6% reported having tried them, and currently, 15.1% use them. Comparing these findings, some studies indicate that 20.9% of adolescents, regardless of sexual orientation, report lifetime use of illicit drugs, and a prevalence of 15.8% of current drug use, such as marijuana, in adolescence^1^ ^,^ ^20^. The high levels found in the LGBT+ population are discussed in the literature through the rejection and discrimination that this population faces, which are closely linked to increased mental health problems, which are recognized as important determinants for substance use among LGBT+ adolescents^21^. In addition, recurring experiences of bullying and oppressive behaviors intensify feelings of isolation and distress, creating an environment conducive to the inappropriate use of substances as a way of coping with or temporarily relieving adversity^22^.

It was found that 31.8% had already smoked, and currently, this frequency is 9.1%. A survey shows that 22.58% of adolescents in the general population reported having tried tobacco, while current use is approximately 6.8%^23^, lower prevalences than those found in the present study, which highlights the greater vulnerability of self-declared LGBT+ adolescents to smoking. Experiences of discrimination based on sexual orientation and gender identity significantly increase the likelihood of tobacco use among LGBT+ youth^24^.

Data on alcohol use in our sample reveal that approximately 77.0% of participants reported having tried alcohol at some point in their lives, a high prevalence when compared to other findings, which found that 52.9% of Brazilian adolescents in the general population had tried alcohol^25^. Current alcohol use was mentioned by 47.2%, which is also a high prevalence when compared to the overall use among adolescents in the general population (25.2%)^26^. A worrying reality of our study population stands out, suggesting that social factors, such as experiencing stigma and challenges related to sexual orientation, may influence this behavior^27^.

In the school environment, bullying is an expression of this structural violence, revealing an alarming reality. Data show that LGBTphobia is the third leading cause of bullying in schools, with 73.0% of LGBT+ students reporting verbal aggression and 36.0% reporting physical aggression^28^. These findings are corroborated by the present study, in which 67.8% (95% CI: 64.7-70.7) of LGBT+ adolescents reported having been victims of bullying, and 37.8% (95% CI: 34.7-40.9) admitted involvement in physical fights.

Violence against LGBT+ adolescents in schools is a significant issue, especially when considering the multiple forms of aggression faced by these individuals and the negative impact of these experiences on their mental health and social well-being. Studies indicate an increase in these episodes, reflecting the structural discrimination that still permeates Brazilian society^29^ ^-^ ^30^.

We observed a high prevalence of cyberbullying among LGBT+ adolescents (11.5%), a significant figure when compared to other international and national realities of adolescents. A study conducted in Yixing, China, reported a general cyberbullying victimization rate of only 2.9% among adolescents, showing a striking difference with the population studied^31^. A comprehensive analysis of data from the 2019 National Student Health Survey (PeNSE, acronym in Portuguese) revealed a prevalence of 13.2% among Brazilian schoolchildren aged 13 to 17^32^. These data suggest that, although cyberbullying is a global issue, its magnitude can vary significantly according to the sociocultural context.

Regarding bullying, 15.2% of participants admitted to having committed acts of this nature, while 7.0% reported engaging in cyberbullying. In this context, factors such as anger, family problems, and the reproduction of violence suffered emerge as pillars of peer conflicts^33^, especially among LGBT+ adolescents, who, despite often being victims of such violence, may also, in some instances, reproduce it as a response to their own experiences of marginalization and suffering^34^.

In addition, 8.0% of participants reported having suffered physical aggression, such as being kicked, pushed, or locked in closed spaces. These behaviors are not only indicative of school violence, but may also reflect an educational environment in which peaceful conflict resolution is not encouraged correctly. Physical fighting can result in both physical and emotional trauma, with lasting consequences for adolescents’ academic development^35^.

Insults related to race, nationality, or skin color were mentioned by approximately 19.0% of adolescents. Racism and xenophobia are forms of psychological violence that have a direct impact on the construction of an individual’s identity and can contribute to increased social isolation and school exclusion^36^; in addition, it should be noted that negative stereotypes in schools contribute significantly to the risk of bullying among racial and ethnic minorities^37^.

Religious comments were reported by 5.5% and obscene jokes or gestures of a sexual nature were reported by 17.3%. Religious comments, in addition to being a form of discrimination, can aggravate identity conflicts, especially in a pluralistic society such as ours^38^. Sexual gestures or comments are subtle forms of sexual violence, often minimized, but with a profound impact on the emotional and psychological development of young people. These jokes or gestures, although not physically invasive, can cause lasting damage to self-esteem and mental health, and they must be identified and addressed in school environments^39^.

LGBT+ adolescents often face bullying related to their appearance, such as criticism of their body shape and facial features, influenced by social beauty standards. In this context, the findings reveal a significant prevalence (44.3%) of adolescents who report being the target of ridicule related to their physical appearance. This type of bullying can have lasting impacts on young people’s self-esteem and well-being, reinforcing the need for educational and support actions to combat stigma and promote acceptance of diversity in schools and society^40^.

Similarly, we note the prevalence of social exclusion, cited as a reason for being excluded from activities on purpose or completely ignored (29.2%). Social attitudes among peers can lead to the exclusion of LGBT+ individuals, with adolescents perceiving that it is more acceptable to exclude gay or lesbian peers compared to heterosexuals^41^. The consequences of social exclusion are severe, with LGBT+ adolescents experiencing higher rates of anxiety, depression, and suicidal thoughts^42^.

Still analyzing the motivations that led LGBT+ adolescents to suffer bullying, it was observed that 13.0% of participants reported that they suffered violence for declaring their sexual orientation. Researchers analyzed a sample of 815 adolescents and identified that those who self-identified as homosexual or bisexual showed significant inverse relationships between homophobic victimization and health-related quality of life, highlighting the harmful effects of this type of bullying^43^. Homophobic bullying is particularly detrimental, resulting in a significant reduction in quality of life and negatively affecting the mood, emotions, and social acceptance of the adolescents affected^42^.

The results of this study highlight the importance of interventions led by health and education professionals, which are essential in preventing violence and promoting inclusive environments for LGBT+ adolescents. Integration between these sectors can enhance the impact of educational and awareness programs, thereby fostering a welcoming and safe school environment. Actions such as training teachers and school administrators, combined with psychological support for students, are fundamental to reducing rates of bullying and discrimination.

It is essential to highlight the limitations inherent in the study design, with possible memory and information biases. However, the relevance of the research lies not only in the use of a comprehensive sample encompassing public and private schools, but also in the use of self-administered questionnaires, which provide greater confidentiality and response accuracy.

The findings provide valuable insights for the development of public policies and interventions to promote equity and health among LGBT+ adolescents, as well as for encouraging health professionals, particularly nurses, to use this information to develop strategies for welcoming this group.

## Conclusion

This study indicated that the socioeconomic profile of LGBT+ adolescents in the RMGV comprises mostly Black individuals, aged 16 to 17, who are public school students and belong to socioeconomic class B. In addition, risky behaviors related to the inappropriate use of condoms and the consumption of psychoactive substances were observed. They report experiences of violence, with high prevalence of bullying, cyberbullying, and social exclusion, motivated mainly by the disclosure of their sexual orientation.

It is undeniable that adolescence is a time of discovery and vulnerability, and for LGBT+ adolescents, these challenges are amplified by stigma and structural barriers. Given this, educators, health professionals, and public administrators must work together to promote more inclusive and safe environments, in which diversity is respected and celebrated. Ensuring the well-being of LGBT+ adolescents is not only a matter of public health, but a commitment to equity and human rights.

The implementation of public policies grounded in this data still faces significant challenges, including sociocultural resistance to including sexual and gender diversity issues in schools and health services. Overcoming these barriers requires coordinated intersectoral actions, continuing professional education, and community engagement.

## Data Availability

Datasets related to this article will be available upon request to the corresponding author.
