# Supplementary material for: Intra-peer violence, socioeconomic and behavioral characteristics of sexual and gender diverse adolescents: a cross-sectional study
Source: Rev Lat Am Enfermagem. 2026 Jul 20;34:e4864. doi: 10.1590/1518-8345.7920.4864 (PMC13387774; doi:10.1590/1518-8345.7920.4864)
Supplement: Translation

# Translation: Violências entre pares, características socioeconômicas e comportamentais de adolescentes com diversidade sexual e de gênero: estudo transversal^*^

**DOI:** 10.1590/1518-8345.7920.4865

**Authors:** Micael Franco Alves, Franciele Marabotti Costa Leite

**Affiliations:** 1 Universidade Federal do Espírito Santo, Vitória, ES, Brasil.

**Keywords:** Adolescentes, Diversidade Sexual, Bullying, Cyberbullying, Violência, Perfil Socioeconômico.

## Abstract

**Objetivo:**

identificar o perfil socioeconômico e comportamental dos adolescentes que se autodeclaram lésbicas, gays, bissexuais, travestis, transexuais e outros, bem como as experiências de violências entre pares e suas motivações.

**Método:**

estudo transversal, descritivo, realizado com 949 adolescentes de 14 a 19 anos, por meio de questionários eletrônicos que foram autopreenchidos e analisados estatisticamente com frequências brutas, relativas e intervalos de confiança de 95%.

**Resultados:**

predominaram adolescentes cisgêneros (81,0%), da classe média (44,7%), raça/cor pretos/pardos (57,4%) e de escolas públicas (67,2%). A prevalência de *bullying* foi de 67,8%, com 13,0% motivados por orientação sexual, e o *cyberbullying* foi identificado em 11,5%. O uso de preservativos foi maior na primeira relação sexual (50,7%) do que na última (40,3%). O uso atual de álcool foi de cerca de 47,2%, o de cigarro de 9,1% e o de drogas foi de 15,1%.

**Conclusão:**

conclui-se que os adolescentes desta pesquisa enfrentam altos índices de violência, exclusão social e vulnerabilidades comportamentais, reforçando a necessidade de estratégias integradas para promoção de ambientes inclusivos e seguros, bem como políticas públicas voltadas à equidade e saúde dessa população.

## Destaques

**(1)** Constatou-se que 67,8% dos adolescentes LGBT+ relataram ser vítimas de *bullying*. **(2)** Treze por cento dos indivíduos relataram sofrer *bullying* motivado por orientação sexual. **(3)** Uso de preservativo foi maior na primeira relação (50,7%) que na última (40,3%). **(4)** Um total de 47,2% dos participantes relatou consumo atual de álcool. **(5)** Dados reforçam a necessidade de políticas para ambientes seguros e inclusivos.

## Introdução

A adolescência é um período crucial de desenvolvimento, caracterizado por transformações físicas, psicológicas e sociais significativas. Nesse estágio, a formação da identidade e a busca pelo equilíbrio diante das mudanças desempenham papéis essenciais na construção do intelecto, da personalidade e das habilidades sociais do indivíduo^1^. A orientação sexual, que é moldada por uma combinação de fatores biológicos, psicológicos, sociais, culturais e familiares, emerge como uma questão central durante essa fase. Compreender essa construção é fundamental para oferecer o apoio adequado aos adolescentes enquanto eles exploram e definem suas identidades^2^.

Entretanto, em um contexto social frequentemente marcado por normas heteronormativas, adolescentes Lésbicas, Gays, Bissexuais, Travestis, Transexuais, *Queer*, Intersexo, Assexuais, Panssexuais e Não Binários (LGBTQIAPN+) enfrentam desafios únicos, como rejeição familiar, intimidação e discriminação. A sigla LGBTQIAPN+* contempla uma ampla diversidade de identidades de gênero e orientações sexuais, incluindo lésbicas (mulheres que se relacionam com mulheres), gays (homens que se relacionam com homens), bissexuais (atração por mais de um gênero), travestis e transexuais (cuja identidade de gênero difere do sexo atribuído ao nascimento), *queer* (identidade dissidente da norma heterocisnormativa), intersexo (pessoas com variações nas características sexuais), assexuais (indivíduos que não sentem atração sexual), pansexuais (atração independente do gênero) e não binários (pessoas que não se identificam exclusivamente como homem ou mulher)^2^. Tais experiências podem comprometer o bem-estar psicológico desses indivíduos e dificultar a formação de uma identidade segura e positiva^3^. Nesse cenário, é imprescindível compreender as dinâmicas sociais e psicológicas que envolvem esses adolescentes, a fim de promover ambientes de apoio, inclusão e proteção.

A violência, em suas diversas formas - física, sexual e psicológica -, é uma questão crítica de saúde pública durante a adolescência. Sua prevalência é amplificada por fatores de risco, como dinâmicas familiares, status socioeconômico e ambientes sociais adversos. Esses fatores precisam ser considerados ao se desenvolver estratégias eficazes de prevenção^4^. Particularmente, o*bullying* dirigido a adolescentes LGBT+ tem se mostrado uma preocupação crescente. Estudos indicam que esses jovens vivenciam uma vitimização mais acentuada, com múltiplas trajetórias de agressão que impactam diretamente sua saúde mental e bem-estar^5^. 

A vulnerabilidade dos adolescentes é intensificada por fatores como identidade de gênero, orientação sexual e raça, criando disparidades adicionais na exposição à violência, especialmente para aqueles com múltiplas identidades marginalizadas^6^.

Neste contexto, os profissionais de saúde e educação desempenham um papel essencial no apoio aos adolescentes LGBT+. Por meio de ações educativas, campanhas de conscientização e o desenvolvimento de espaços seguros e inclusivos, esses profissionais podem ajudar a mitigar os impactos da discriminação e promover a saúde mental e o bem-estar. Estratégias integradas entre os setores de saúde e educação são fundamentais para a prevenção de violências e a construção de uma sociedade mais equitativa^7^.

Diante desse cenário, o objetivo deste artigo é identificar o perfil socioeconômico e comportamental dos adolescentes que se autodeclaram lésbicas, gays, bissexuais, travestis, transexuais e outros, bem como as experiências de violências entre pares e suas motivações.

## Método

### Delineamento do estudo

Trata-se de um estudo transversal descritivo, realizado em 63 escolas públicas e privadas de ensino médio localizadas na Região Metropolitana da Grande Vitória (RMGV), Espírito Santo, abrangendo os municípios de Cariacica, Fundão, Guarapari, Serra, Viana, Vila Velha e Vitória.

### Local 

O estudo foi realizado em 38 escolas públicas e 25 privadas da RMGV, Espírito Santo, Brasil, que foram selecionadas de forma aleatória, totalizando 63 escolas. Atualmente, essa região conta com 171 escolas com ensino médio regular, públicas e/ou privadas; assim, as escolas que realizamos o estudo compreendem 36,8%. 

### Período

A coleta de dados ocorreu entre março e dezembro de 2023.

### População

A população total entrevistada foi de 4614 adolescentes matriculados no ensino médio, sendo o recorte para o presente estudo de 949 alunos que se autodeclararam LGBT+. Como critérios de exclusão, foram adotados os déficits cognitivos que impedissem a compreensão do questionário. Para o presente estudo, entendem-se por adolescentes as pessoas entre 10 e 19 anos, conforme a Organização Mundial da Saúde^8^. 

### Definição da amostra

Esse estudo é um recorte de uma pesquisa maior intitulada Pesquisa Estadual de Saúde do Escolar Capixaba (PESC), em que, na amostra, garantindo representatividade comparativa entre as redes pública e privada, foi utilizada a técnica de estratificação em Rede pública e Rede privada. Em seguida, amostras simples foram obtidas para cada estrato, sendo posteriormente corrigidas para amostras complexas, utilizando o *software* SPSS 26, assegurando um intervalo de confiança de 95% e um erro máximo de 5%. Além disso, para cada estrato municipal, foram estabelecidos parâmetros de 95% de confiança e erro máximo de 10%. A amostragem foi realizada por meio de conglomerados e estratificação em 3 estratos diferentes, usando o percentual de 95% de confiança e erro máximo de 5%. Para cada estrato de município, o erro máximo foi de 10%. 

### Variáveis do estudo

As variáveis analisadas incluíram: socioeconômicas: idade (14/15, 16/17, 18/19 anos); identidade de gênero, em que cisgênero foi classificada como identidade de gênero correspondente àquela atribuída no nascimento, classificada em cisgênero ou não-cisgênero; raça/cor da pele (branca, preta, amarela, parda, indígena); está em algum relacionamento (não, sim); tipo de escola (pública, privada); classificação socioeconômica (A, B, C, D/E) - para essa variável utilizamos o critério da Associação Brasileira de Empresas de Pesquisa^9^; religião (nenhuma, católica, evangélica, espírita, raiz africana, outra); possui trabalho/estágio (não, sim); status dos pais (vivem juntos, vivem separados, um deles já faleceu - ou os dois); comportamentais: já teve relação sexual (não, sim); uso de preservativo na primeira (não, sim); uso de preservativo na última relação sexual (não, sim); experimentação de drogas (não, sim); uso atual de drogas (não, sim); experimentação de cigarro (não, sim); uso atual de cigarro (não, sim); experimentação de bebida alcoólica (não, sim); uso atual de bebida alcoólica (não, sim); violência entre pares: vítima de *bullying* (não, sim); praticou *bullying* (não, sim); vítima de *cyberbullying* (não, sim); praticou *cyberbullying* (não, sim); frequência com que se envolveu em brigas físicas (nunca se envolveu, envolveu-se uma ou mais vezes); tipos de *bullying* sofridos: batiam/esbarravam/chutado/trancado em locais fechados/empurrado (não, sim); era zoado por causa da raça, nacionalidade ou cor de pele (não, sim); era zoado por causa da religião (não, sim); era zoado por meio de brincadeiras ou comentários de cunho sexual ou gestos obscenos (não, sim); era excluído de atividades de propósito ou completamente ignorado (não, sim); era zoado por causa da aparência do corpo ou rosto (não, sim); sofria outra forma de *bullying* (não, sim); motivação para o *bullying*: cor/raça (não, sim); religião (não, sim); aparência do rosto (não, sim); aparência do corpo (não, sim); orientação sexual (não, sim); região de origem (não, sim); outros (não, sim).

### Instrumentos utilizados para coleta das informações

A coleta de dados foi realizada por meio de questionários eletrônicos autopreenchidos em *tablets* por meio do sistema RedCAP (*Research Electronic Data Capture*), compostos por blocos de caracterização socioeconômica e comportamental. Além disso, as experiências de violências entre pares e suas motivações foram rastreadas por meio de instrumento baseado no *Adverse Childhood Experiences International Questionnaire* (ACE-IQ), adaptado e validado para uso no Brasil^10^.

### Coleta de dados

A coleta de dados foi conduzida por uma equipe responsável, treinada e supervisionada para assegurar a qualidade e integridade das informações. Antes do início da pesquisa, foi solicitada e obtida autorização da Secretaria de Estado da Educação (SEDU) para a realização do estudo nas escolas públicas sorteadas. Em seguida, estabeleceu-se contato prévio com as instituições públicas, assim como as escolas privadas, para apresentação da proposta em seminário realizado em março de 2023, onde aconteceu a participação de aproximadamente 80% das escolas selecionadas para a pesquisa. Na presente ocasião, foram expostos os objetivos e procedimentos da pesquisa e entregues os Termos de Consentimento Livre e Esclarecido (TCLE) aos representantes das instituições. Para as escolas ausentes, a equipe realizou visitas presenciais, garantindo adesão de 100% das unidades sorteadas. Posteriormente, foi feito novo contato para recolhimento nas escolas do TCLE e agendamento da coleta de dados, que acontecia em sala de aula e/ou auditório, conforme disponibilidade da escola. Importante destacar que, no momento da coleta de dados, a equipe distribuía um *tablet* para cada aluno participante e realizava as orientações para o uso do material. O tempo médio de preenchimento do questionário era cerca de cinquenta minutos.

### Tratamento e análise dos dados

As análises estatísticas descritivas foram conduzidas mediante apresentação de frequência bruta, relativa e intervalos de confiança de 95% (IC95%). Todas as análises foram realizadas por meio do programa estatístico Stata 17.0. 

### Aspectos éticos

Este estudo foi aprovado pelo Comitê de Ética em Pesquisa da Universidade Federal do Espírito Santo, em conformidade com a Resolução 466/2012 do Conselho Nacional de Saúde. Pais e/ou responsáveis pelos adolescentes participantes assinaram o Termo de Consentimento Livre e Esclarecido (TCLE) e, os adolescentes, o Termo de Assentimento Livre e Esclarecido (TALE). Ressalta-se que essa pesquisa foi financiada pela Fundação de Amparo à Pesquisa do Espírito Santo (FAPES) sob o número de processo CAAE 65545622.5.0000.5060.

## Resultados

As características socioeconômicas dos escolares autodeclarados LGBT+ da RMGV (Tabela 1) mostraram que a maioria se declarou como negra (pardos - 38,9%; pretos - 18,5%). Predominaram adolescentes de 16 a 17 anos (65,5%), que não estavam em um relacionamento (69,0%), cisgêneros (81,0%) e frequentavam escolas públicas (67,2%).

Nota-se que 44,7% pertenciam às classes B e 45,3% não tinham religião. Por fim, 76,7% não trabalhavam e, no *status* dos pais, 47,1% viviam separados.

**Tabela 1:** Características socioeconômicas dos escolares autodeclarados LGBT+. Vitória, ES, Brasil, 2023

Variável	N*	%^†^	IC95%^‡^
Faixa etária (anos)			
14 a 15	204	21,5	19,0-24,2
16 a 17	621	65,5	62,4-68,5
18 a 19	123	13,0	11,0-15,2
Identidade de gênero			
Cisgênero	769	81,0	78,4-83,4
Não cisgênero	180	19,0	16,6-21,6
Raça/Cor			
Branca (o)	380	40,0	37,0-43,2
Preta (o)	175	18,5	16,1-21,0
Amarela (o)	15	1,6	0,9-2,0
Parda (o)	369	38,9	35,8-42,0
Indígena	10	1,0	0,5-2,0
Está em algum relacionamento			
Não	655	69,0	66,0-71,8
Sim	294	31,0	28,1-33,9
Escola pública ou privada			
Pública	638	67,2	64,1-70,1
Privada	311	32,8	29,9-34,6
Classificação socioeconômica			
A	154	16,3	14,0-18,7
B	424	44,7	41,6-47,9
C	317	33,5	30,5-36,5
D/E	53	5,5	4,2-7,2
Religião			
Nenhuma	429	45,3	42,0-48,4
Católica	167	17,6	15,3-20,1
Evangélica	215	22,6	20,1-25,4
Espírita	54	5,7	4,4-7,4
Raiz Africana	48	5,0	3,8-6,6
Outra	36	3,8	2,7-5,2
Possui trabalho/estágio			
Não	728	76,7	74,0-79,3
Sim	221	23,3	20,7-26,0
*Status* **dos pais**			
Vivem juntos	424	45,1	41,9-48,3
Vivem separados	443	47,1	43,9-50,3
Um deles já faleceu (ou os dois)	73	7,8	6,2-9,6

*N = Frequência absoluta; ^†^% = Prevalência; ^‡^IC95% = Intervalo de confiança de 95%

As características comportamentais dos escolares autodeclarados LGBT+ da RMGV são apresentadas na Tabela 2. Em relação à experiência sexual, a maioria (56,6%) nunca teve relação sexual, enquanto 43,4% afirmaram já ter tido. O uso de preservativo na primeira relação sexual foi apontado por cerca de 51,0% dos adolescentes (IC95%: 45,9-55,5); todavia, na última relação sexual, aproximadamente 40,0% afirmaram fazerem uso (IC95%: 44,4-54,1). Quanto ao uso de drogas ao longo da vida, 33,6% já usaram, e, atualmente, 15,1% fazem uso. Verifica-se que 31,8% já fumaram e atualmente essa frequência é de 9,1%. Em relação ao uso de bebidas alcoólicas ao longo da vida, 77,2% já consumiram e 47,2% fazem uso no momento presente.

**Tabela 2:** Características comportamentais dos escolares autodeclarados LGBT+. Vitória, ES, Brasil, 2023

Variável	N*	%^†^	IC95%^‡^
Já teve relação sexual			
Não	537	56,6	53,4-59,7
Sim	412	43,4	40,3-46,6
Uso de preservativo na primeira relação sexual			
Não	203	49,3	44,4-54,1
Sim	209	50,7	45,9-55,5
Uso de preservativo na última relação sexual			
Não	246	59,7	54,9-64,3
Sim	166	40,3	35,7-45,1
Experimentou drogas			
Não	630	66,4	63,3-69,3
Sim	319	33,6	30,7-36,7
Uso de drogas atualmente			
Não	805	84,8	82,4-87,0
Sim	144	15,2	13,0-17,6
Experimentou cigarro			
Não	647	68,2	65,1-71,0
Sim	302	31,8	29,0-34,8
Uso atual de cigarro			
Não	863	90,9	89,0-92,6
Sim	86	9,1	7,3-11,0
Experimentou bebida alcoólica			
Não	216	22,8	20,2-25,6
Sim	731	77,2	74,4-79,7
Uso atual de bebida alcoólica			
Não	500	52,8	49,6-56,0
Sim	447	47,2	44,0-50,4

*N = Frequência absoluta; ^†^% = Prevalência; ^‡^IC95% = Intervalo de confiança de 95%

No que diz respeito à prevalência de violência entre pares (Tabela 3), 67,8% dos escolares LGBT+ relataram já terem sido vítimas de *bullying* e 11,5% afirmaram ter vivenciado *cyberbullying*. Em relação à prática de *bullying*, 15,2% dos participantes admitiram já ter cometido atos dessa natureza, enquanto 7,0% relataram a prática de *cyberbullying*. Além disso, cerca de 38,0% declararam envolvimento em brigas físicas pelo menos uma vez.

**Tabela 3:** Prevalência de *bullying* e *cyberbullying* entre os escolares autodeclarados como LGBT+. Vitória, ES, Brasil, 2023

Variável	N*	%^†^	IC95%^‡^
Vítima de *bullying*			
Não	306	32,2	29,3-35,3
Sim	643	67,8	64,7-70,7
Praticou *bullying*			
Não	803	84,8	82,4-86,9
Sim	144	15,2	13,1-17,6
Vítima de *cyberbullying*			
Não	838	88,5	86,3-90,4
Sim	109	11,5	9,6-13,7
Praticou *cyberbullying*			
Não	881	93,0	91,2-94,5
Sim	66	7,0	5,5-8,8
Frequência com que se envolveu em briga física			
Nunca se envolveu	569	62,3	59,1-65,3
Envolveu-se uma ou mais vezes	345	37,7	34,7-40,9

*N = Frequência absoluta; ^†^% = Prevalência; ^‡^IC95% = Intervalo de confiança de 95%

Quanto aos tipos de *bullying* (Tabela 4), observa-se que 8,0% dos participantes relataram terem sofrido agressões físicas, como serem chutados, empurrados ou trancados em locais fechados. Insultos relacionados à raça, nacionalidade ou cor de pele foram mencionados por aproximadamente 19,0%. Comentários de cunho religioso foram relatados por 5,5% e brincadeiras ou gestos obscenos de cunho sexual foram apontados por 17,3%. Além disso, 29,2% relataram serem excluídos de atividades ou completamente ignorados, enquanto 44,3% sofreram insultos relacionados à aparência física e outras formas de *bullying* foram identificadas por 12,9%.

**Tabela 4:** Tipos de *bullying* praticados contra os escolares autodeclarados como LGBT+. Vitória, ES, Brasil, 2023

Variável	N*	%^†^	IC95%^‡^
Batiam/Esbarravam/Chutado/Trancado em locais fechados/Empurrado			
Não	873	92,0	90,0-93,5
Sim	76	8,0	6,4-10,0
Era zoado (a) (debochavam) por causa da raça, nacionalidade ou cor de pele			
Não	770	81,1	78,5-83,5
Sim	179	18,9	16,5-21,5
Era zoado (a) (debochavam) por causa da religião			
Não	897	94,5	92,8-95,8
Sim	52	5,5	4,2-7,1
Era zoado (a) (debochavam) por meio de brincadeiras ou comentários de cunho sexual ou gestos obscenos			
Não	785	82,7	80,1-85,0
Sim	164	17,3	15,0-19,9
Era excluído (a) de atividades de propósito ou completamente ignorado (a)			
Não	672	70,8	67,8-73,6
Sim	277	29,2	26,4-32,2
Era zoado (a) (debochavam) por causa da aparência do corpo ou rosto			
Não	529	55,7	52,6-58,9
Sim	420	44,3	41,1-47,4
Sofria outra forma de *bullying*			
Não	827	87,1	84,8-89,1
Sim	122	12,9	10,9-15,1

*N = Frequência absoluta; ^†^% = Prevalência; ^‡^IC95% = Intervalo de confiança de 95%

A análise das motivações para o *bullying* entre adolescentes LGBT+ (Tabela 5) revelou que a cor ou raça foi mencionada por 5,0% dos participantes, enquanto a religião foi relatada por 4,2%. Em relação à aparência do rosto, 9,1% dos adolescentes relataram ter sofrido *bullying* por esse motivo e 10,4% indicaram a aparência do corpo como fator motivador. Vale destacar que a orientação sexual apresentou a maior prevalência entre os motivos apresentados, sendo citada por 13,0% dos participantes.

**Tabela 5:** Motivações para ocorrência do *bullying* praticado contra os escolares autodeclarados como LGBT+. Vitória, ES, Brasil, 2023

Variável	N*	%^†^	IC95%^‡^
Cor/Raça			
Não	902	95,0	93,4-96,2
Sim	47	5,0	3,7-6,5
Religião			
Não	909	95,8	94,3-96,8
Sim	40	4,2	3,1-5,6
Aparência do rosto			
Não	863	90,9	88,9-92,6
Sim	86	9,1	7,4-11,0
Aparência do corpo			
Não	850	89,6	87,4-91,3
Sim	99	10,4	8,6-12,5
Orientação sexual			
Não	826	87,0	84,7-89,0
Sim	123	13,0	10,9-15,2
Região de origem			
Não	922	97,1	95,8-98,0
Sim	27	2,8	1,9-4,1
Outros			
Não	862	90,8	88,8-92,5
Sim	87	9,2	7,4-11,1

*N = Frequência absoluta; ^†^% = Prevalência; ^‡^IC95% = Intervalo de confiança de 95%

## Discussão

Os dados socioeconômicos apresentados revelam aspectos importantes sobre o perfil dos escolares autodeclarados LGBT+ do Espírito Santo. Observa-se que 65,5% estavam na faixa etária de 16 e 17 anos, predominantemente cisgêneros (81,0%), o que reflete a tendência majoritária em muitas sociedades, embora uma parcela significativa (19,0%) tenha se identificado como não cisgêneros, destacando a diversidade de identidades de gênero dentro desse grupo^11^. Além disso, 69,0% dos participantes não estavam em um relacionamento, o que pode indicar um padrão de relacionamentos ou uma fase da vida em que a maioria ainda não se envolveu em relacionamentos formais, algo comum em faixas etárias mais jovens^12^. 

Em termos de religião, 45,3% dos adolescentes se declararam sem religião. Muitos adolescentes LGBT+ vivenciam um conflito entre suas identidades sexuais e religiosas, o que frequentemente resulta em sentimentos de desconexão de suas comunidades religiosas^11^. Relatam experiências de conexões iniciais com sua fé, mas posteriormente passam a questionar suas crenças devido à sua orientação sexual^13^.

Ao comparar os dados obtidos na pesquisa com outros estudos do Brasil, observa-se que a prevalência de iniciação sexual na presente pesquisa entre os adolescentes da amostra é consideravelmente mais alta (43,4%). Estudo realizado no Piauí, com adolescentes sem recorte por orientação sexual, indicou uma prevalência de 24,2%^14^ e, em Curitiba, também com a população geral de adolescentes, a prevalência foi de 22,9%^15^. Esses dados sugerem que a iniciação sexual é um fenômeno presente na adolescência LGBT+, tendo a orientação sexual como fator que pode influenciar a idade de início, reforçando a importância de programas de educação sexual para este público. 

Neste estudo, aproximadamente 51,0% dos participantes relataram uso de preservativo na primeira relação sexual, achado superior ao identificado em pesquisa com adolescentes da população geral (24,7%)^16^. Por outro lado, na última relação sexual, a prevalência de uso foi de 40,3%, percentual inferior ao encontrado em outro estudo realizado com adolescentes da população geral (68,8%)^17^. Esses achados sugerem que adolescentes LGBT+ tendem a adotar maior proteção no início da vida sexual, motivados principalmente pela prevenção de ISTs, desconfiança ou imposição do(a) parceiro(a). Contudo, a redução na adesão ao preservativo nas relações subsequentes pode estar relacionada à confiança no parceiro, preferências pessoais, esquecimento ou indisponibilidade do método^18^.

Diante do exposto, é importante chamarmos atenção para o trabalho de educação em saúde que precisa ser realizado no âmbito escolar por profissionais da saúde, justamente para que a adesão aos métodos contraceptivos seja estendida para além da primeira relação sexual, pois, por meio desta ação, há melhora na compreensão dos adolescentes sobre saúde sexual, levando a boas atitudes e comportamentos em relação às IST^19^. 

Quanto às drogas, 33,6% declararam já terem experimentado, e, atualmente, 15,1% fazem uso. Comparando esses achados, há estudos que apontam 20,9% dos adolescentes, sem distinção de orientação sexual, relatando uso de drogas ilícitas ao longo da vida, e uma prevalência de 15,8% do uso atual de droga, como a maconha, na adolescência^1^ ^,^ ^20^. Os elevados níveis encontrados na população LGBT+ são discutidos na literatura à luz da rejeição e discriminação que essa população enfrenta, que estão intimamente ligados ao aumento de problemas de saúde mental, os quais são reconhecidos como determinantes importantes para o uso de substâncias entre adolescentes LGBT+^21^. Além disso, as experiências recorrentes de *bullying* e comportamentos opressivos intensificam sentimentos de isolamento e angústia, criando um ambiente propício para o uso inadequado de substâncias como forma de enfrentamento ou alívio temporário das adversidades^22^.

Verifica-se que 31,8% já fumaram e, atualmente, essa frequência é de 9,1%. Uma pesquisa aponta que 22,58% dos adolescentes da população geral relataram ter experimentado tabaco, enquanto o uso atual é de aproximadamente 6,8%^23^, prevalências menores do que as encontradas na presente pesquisa, o que evidencia a maior vulnerabilidade dos adolescentes autodeclarados LGBT+ ao tabagismo. As experiências de discriminação com base na orientação sexual e identidade de gênero aumentam significativamente a probabilidade de uso de tabaco entre jovens LGBT+^24^. 

Os dados sobre o uso de álcool em nossa amostra revelam aproximadamente 77,0% dos participantes relatando a experimentação de álcool alguma vez na vida, prevalência alta quando comparamos o resultado com outros achados, que constataram 52,9% de experimentação por parte de adolescentes brasileiros da população geral^25^. Já o uso atual de álcool foi mencionado por 47,2%, prevalência também alta quando comparamos com o uso global de adolescentes da população geral (25,2%)^26^. Destaca-se uma realidade preocupante da nossa população estudada, sugerindo que fatores sociais, como a vivência de estigmas e desafios relacionados à orientação sexual, podem influenciar esse comportamento^27^.

No ambiente escolar, o *bullying* se configura como uma expressão dessa violência estrutural, revelando uma realidade alarmante. Dados apontam que a LGBTfobia é a terceira maior causa de *bullying* nas escolas, com 73,0% dos estudantes LGBT+ relatando agressões verbais e 36,0% relatando agressões físicas^28^. Esses achados são corroborados pelo presente estudo, no qual 67,8% (IC95%: 64,7-70,7) dos adolescentes LGBT+ relataram ter sido vítimas de *bullying* e 37,8% (IC95%: 34,7-40,9) admitiram envolvimento em brigas físicas.

A violência contra adolescentes LGBT+ nas escolas representa uma questão relevante, especialmente ao se considerarem as múltiplas formas de agressão enfrentadas por esses indivíduos e o impacto negativo dessas experiências sobre sua saúde mental e bem-estar social. Estudos indicam um crescimento desses episódios, refletindo a discriminação estrutural que ainda permeia a sociedade brasileira^29^ ^-^ ^30^. 

Observamos uma alta prevalência de *cyberbullying* entre adolescentes LGBT+ (11,5%), um valor significativo ao compararmos com outras realidades internacionais e nacionais de adolescentes. Um estudo realizado em Yixing, na China, relatou uma taxa de vitimização por *cyberbullying* geral de apenas 2,9% entre adolescentes, evidenciando uma diferença marcante com a população estudada^31^. Já uma análise abrangente dos dados da Pesquisa Nacional de Saúde do Estudante (PeNSE) de 2019 revelou uma prevalência de 13,2% entre escolares brasileiros de 13 a 17 anos^32^. Esses dados sugerem que, embora o *cyberbullying* seja uma questão global, sua magnitude pode variar significativamente de acordo com o contexto sociocultural. 

Em relação à prática de *bullying*, 15,2% dos participantes admitiram já ter cometido atos dessa natureza, enquanto 7,0% relataram a prática de *cyberbullying*. Nesse contexto, fatores como raiva, problemas familiares e a reprodução de uma violência sofrida emergem como pilares dos conflitos entre pares^33^, especialmente entre adolescentes LGBT+, que, apesar de muitas vezes serem vítimas dessa violência, também podem, em certos casos, reproduzi-la como uma resposta a suas próprias experiências de marginalização e sofrimento^34^.

Ademais, 8,0% dos participantes relataram terem sofrido agressões físicas, como serem chutados, empurrados ou trancados em locais fechados. Esses comportamentos não só são indicativos de violência escolar, mas também podem ser reflexos de um ambiente educacional em que a resolução de conflitos de forma pacífica não é devidamente incentivada. A prática de brigas físicas pode resultar em traumas tanto físicos quanto emocionais, com consequências duradouras no desenvolvimento acadêmico dos adolescentes^35^.

Insultos relacionados à raça, nacionalidade ou cor de pele foram mencionados por aproximadamente 19,0% dos adolescentes. O racismo e a xenofobia são formas de violência psicológica que têm impacto direto na construção da identidade do indivíduo e podem contribuir para o aumento do isolamento social e da exclusão escolar^36^; além disso, destaca-se que os estereótipos negativos nas escolas contribuem significativamente para o risco de bullying entre minorias raciais e étnicas^37^.

Comentários de cunho religioso foram relatados por 5,5% e brincadeiras ou gestos obscenos de cunho sexual foram apontados por 17,3%. Comentários de cunho religioso, além de serem uma forma de discriminação, podem agravar conflitos identitários, especialmente em uma sociedade plural como a nossa^38^. Já os gestos ou comentários de cunho sexual são formas sutis de violência sexual, muitas vezes minimizadas, mas com impacto profundo no desenvolvimento emocional e psicológico dos jovens. Essas brincadeiras ou gestos, embora não fisicamente invasivos, podem gerar danos duradouros à autoestima e à saúde mental, sendo essencial que sejam identificados e abordados em ambientes escolares^39^.

Adolescentes LGBT+ frequentemente enfrentam *bullying* relacionado à sua aparência, como críticas ao formato corporal e características faciais, influenciadas pelos padrões de beleza social. Neste contexto, os achados revelam uma prevalência significativa (44,3%) de adolescentes que relatam ser alvo de zombarias relacionadas à aparência física. Esse tipo de *bullying* pode ter impactos duradouros na autoestima e bem-estar dos jovens, reforçando a necessidade de ações educativas e de apoio para combater o estigma e promover a aceitação da diversidade nas escolas e na sociedade^40^.

De igual modo, chama-nos a atenção a prevalência de exclusão social, dita como motivação em ser excluído(a) de atividades de propósito ou completamente ignorado(a) (29,2%). As atitudes sociais entre colegas podem levar à exclusão de indivíduos LGBT+, com os adolescentes percebendo que é mais aceitável excluir colegas gays ou lésbicas em comparação com os heterossexuais^41^. As consequências da exclusão social são graves, com adolescentes LGBT+ experimentando taxas mais altas de ansiedade, depressão e pensamentos suicidas^42^.

Ainda analisando as motivações que levaram os adolescentes LGBT+ a sofrerem *bullying*, observa-se que 13,0% dos participantes relataram que sofreram violência por ter declarado a sua orientação sexual. Pesquisadores analisaram uma amostra de 815 adolescentes e identificaram que aqueles que se autodeclararam homossexuais ou bissexuais apresentaram relações inversas significativas entre vitimização homofóbica e qualidade de vida relacionada à saúde, evidenciando os efeitos prejudiciais deste tipo de *bullying* ^43^. O *bullying* homofóbico é particularmente danoso, resultando em uma redução significativa da qualidade de vida, afetando negativamente humor, emoções e aceitação social dos adolescentes afetados^42^.

Os resultados deste estudo destacam a importância de intervenções direcionadas pelos profissionais de saúde e educação, que são essenciais na prevenção de violências e na promoção de ambientes inclusivos para adolescentes LGBT+. A integração entre esses setores pode potencializar o impacto de programas educativos e de conscientização, favorecendo a construção de um ambiente escolar acolhedor e seguro. Ações como treinamento de professores e gestores escolares, aliadas ao suporte psicológico aos alunos, são fundamentais para reduzir os índices de *bullying* e discriminação.

É importante destacar as limitações inerentes ao desenho do estudo, com possíveis vieses de memória e informação. Todavia, a relevância da pesquisa está não apenas na utilização de uma amostra abrangente, contemplando escolas públicas e privadas, mas também no uso de questionários autoaplicados que permitem maior confidencialidade e precisão nas respostas. 

Os achados fornecem subsídios valiosos para o desenvolvimento de políticas públicas e intervenções voltadas à promoção da equidade e saúde da população adolescente LGBT+, além de estimular profissionais da saúde, sobretudo da enfermagem, a utilizar destas informações para o desenvolvimento de estratégias de acolhimento para este grupo. 

## Conclusão 

Este estudo evidenciou que o perfil socioeconômico dos adolescentes LGBT+ da RMGV compreende em sua maioria negros, na faixa etária de 16 a 17 anos, estudantes de escola pública e pertencentes à classe socioeconômica B. Além disso, observa-se a presença de comportamentos de risco relacionados ao uso inadequado de preservativos e consumo de substâncias psicoativas. Relatam experiências de violência, com altas prevalências de *bullying*, *cyberbullying* e exclusão social, em sua maioria sendo motivada pelo relato de declaração da sua orientação sexual.

É inegável que a adolescência é uma fase de descobertas e vulnerabilidades, e para adolescentes LGBT+, esses desafios são ampliados por estigmas e barreiras estruturais. Diante disso, é essencial que educadores, profissionais de saúde e gestores públicos atuem de forma integrada, promovendo ambientes mais inclusivos e seguros, nos quais a diversidade seja respeitada e celebrada. Garantir o bem-estar dos adolescentes LGBT+ não é apenas uma questão de saúde pública, mas um compromisso com a equidade e os direitos humanos.

A implementação de políticas públicas baseadas nesses dados ainda enfrenta desafios significativos, como a resistência sociocultural à inclusão de questões de diversidade sexual e de gênero no ambiente escolar e nos serviços de saúde. Superar essas barreiras exige ações intersetoriais coordenadas, formação continuada de profissionais e engajamento comunitário.

## Data Availability

Os conjuntos de dados relacionados a este artigo estarão disponíveis mediante solicitação ao autor correspondente.

# Translation: Violencia entre iguales, características socioeconómicas y conductuales de adolescentes con diversidad sexual y de género: un estudio transversal^*^

**DOI:** 10.1590/1518-8345.7920.4863

**Authors:** Micael Franco Alves, Franciele Marabotti Costa Leite

**Affiliations:** 1 Universidade Federal do Espírito Santo, Vitória, ES, Brasil.

**Keywords:** Adolescentes, Diversidad de Género, Bullying, Ciberacoso, Violencia, Perfil Socioeconómico.

## Abstract

**Objetivo:**

identificar el perfil socioeconómico y conductual de adolescentes que se autodeclaran lesbianas, gays, bisexuales, travestis, transexuales y otros, así como las experiencias de violencia entre iguales y sus motivaciones.

**Método:**

estudio transversal y descriptivo realizado con 949 adolescentes de entre 14 y 19 años, utilizando cuestionarios electrónicos autocompletados y analizados estadísticamente con frecuencias brutas y relativas e intervalos de confianza del 95%.

**Resultados:**

predominó adolescentes cisgénero (81,0%), clase media (44,7%), raza/color negro/marrón (57,4%) y escuelas públicas (67,2%). La prevalencia del acoso escolar fue del 67,8%, con un 13,0% motivado por la orientación sexual, y el ciberacoso se identificó en un 11,5%. El uso de preservativos fue mayor en la primera relación sexual (50,7%) que en la anterior (40,3%). El consumo actual de alcohol fue de aproximadamente el 47,2%, los cigarrillos del 9,1% y los de drogas del 15,1%.

**Conclusión:**

se concluye que los adolescentes en esta investigación enfrentan altas tasas de violencia, exclusión social y vulnerabilidades conductuales, lo que refuerza la necesidad de estrategias integradas para promover entornos inclusivos y seguros, así como políticas públicas orientadas a la equidad y la salud de esta población.

## Destacados

**(1)** Se encontró que el 67,8% de los adolescentes LGBT+ reportaron haber sido víctimas de acoso escolar. **(2)** El trece por ciento de las personas reportó haber sido acosada por su orientación sexual. **(3)** El uso de preservativo fue mayor en la primera relación sexual (50,7%) que en la anterior (40,3%). **(4)** Un total del 47,2% de los participantes reportó consumo actual de alcohol. **(5)** Los datos refuerzan la necesidad de políticas para entornos seguros e inclusivos.

## Introducción

La adolescencia es un periodo crucial de desarrollo, caracterizado por transformaciones físicas, psicológicas y sociales significativas. En esta etapa, la formación de la identidad y la búsqueda del equilibrio ante los cambios desempeñan papeles esenciales en la construcción del intelecto, la personalidad y las habilidades sociales del individuo^1^. La orientación sexual, que está moldeada por una combinación de factores biológicos, psicológicos, sociales, culturales y familiares, emerge como un tema central durante esta fase. Comprender esta construcción es esencial para ofrecer un apoyo adecuado a los adolescentes mientras exploran y definen sus identidades^2^.

Sin embargo, en un contexto social a menudo marcado por normas heteronormativas, adolescentes lesbianas, gays, bisexuales, travestis, transexuales, *queer*, intersexuales, asexuales, pansexuales y no binarios (LGBTQIAPN+) se enfrentan a desafíos únicos, como el rechazo familiar, la intimidación y la discriminación. El acrónimo LGBTQIAPN+* abarca una amplia diversidad de identidades de género y orientaciones sexuales, incluyendo lesbianas (mujeres que tienen relaciones con mujeres), gays (hombres que tienen relaciones con hombres), bisexuales (atracción por más de un género), travestis y transexuales (cuya identidad de género difiere del sexo asignado al nacer), *queer* (disidente de identidad respecto a la norma heterocisnormativa), intersexuales (personas con variaciones en las características sexuales), asexual (personas que no sienten atracción sexual), pansexual (atracción independientemente del género) y no binaria (personas que no se identifican exclusivamente como hombre o mujer)^2^. Estas experiencias pueden comprometer el bienestar psicológico de estas personas y dificultar la formación de una identidad segura y positiva^3^. En este escenario, es esencial comprender las dinámicas sociales y psicológicas que involucran a estos adolescentes, para promover entornos de apoyo, inclusión y protección.

La violencia, en sus diversas formas - física, sexual y psicológica - es un problema crítico de salud pública durante la adolescencia. Su prevalencia se amplifica por factores de riesgo como la dinámica familiar, el estatus socioeconómico y entornos sociales adversos. Estos factores deben tenerse en cuenta al desarrollar estrategias de prevención efectivas^4^. En particular, el acoso dirigido a adolescentes LGBT+ ha sido una preocupación creciente. Los estudios indican que estos jóvenes experimentan una victimización más acentuada, con múltiples trayectorias de agresión que afectan directamente a su salud mental y bienestar^5^.

La vulnerabilidad de los adolescentes se intensifica por factores como la identidad de género, la orientación sexual y la raza, lo que genera disparidades adicionales en la exposición a la violencia, especialmente entre quienes tienen múltiples identidades marginadas^6^.

En este contexto, los profesionales de la salud y la educación desempeñan un papel esencial en el apoyo a los adolescentes LGBT+. A través de acciones educativas, campañas de concienciación y el desarrollo de espacios seguros e inclusivos, estos profesionales pueden ayudar a mitigar los impactos de la discriminación y promover la salud mental y el bienestar. Las estrategias integradas entre los sectores de salud y educación son fundamentales para la prevención de la violencia y la construcción de una sociedad más equitativa^7^ ^).^

En vista de este escenario, el objetivo de este artículo es identificar el perfil socioeconómico y conductual de adolescentes que se autodeclaran lesbianas, gays, bisexuales, travestis, transexuales y otros, así como las experiencias de violencia entre iguales y sus motivaciones.

## Método

### Diseño del estudio

Se trata de un estudio descriptivo transversal, realizado en 63 institutos públicos y privados situados en la Región Metropolitana de la Gran Vitória (RMGV), Espírito Santo, que abarca los municipios de Cariacica, Fundão, Guarapari, Serra, Viana, Vila Velha y Vitória.

### Local 

El estudio se llevó a cabo en 38 escuelas públicas y 25 privadas de la RMGV, Estado de Espírito Santo, Brasil, que fueron seleccionadas aleatoriamente, sumando un total de 63 escuelas. Actualmente, esta región cuenta con 171 escuelas con institutos regulares, públicos y/o privados; Así, las escuelas que realizamos el estudio representan el 36,8%.

### Periodo

La recopilación de datos tuvo lugar entre marzo y diciembre de 2023.

### Población

La población total entrevistada fue de 4.614 adolescentes matriculados en la escuela secundaria, y el enfoque del presente estudio fueron 949 estudiantes que se declararon LGBT+. Como criterios de exclusión, se adoptaron déficits cognitivos que impedían la comprensión del cuestionario. Según el presente estudio, se entiende que los adolescentes tienen entre 10 y 19 años, según la Organización Mundial de la Salud^8^.

### Definición de la muestra

Este estudio es un extracto de un estudio más amplio titulado Encuesta Estatal de Salud del Escolar Capixaba (PESC), en el que, en la muestra, para asegurar la representatividad comparativa entre redes públicas y privadas, se utilizó la técnica de estratificación en redes públicas y privadas. Luego, se obtuvieron muestras simples para cada estrato, que posteriormente se corrigieron para muestras complejas utilizando el *software* SPSS 26, asegurando un intervalo de confianza del 95% y un error máximo del 5%. Además, para cada estrato municipal, se establecieron parámetros con un 95% de confianza y un error máximo del 10%. El muestreo se realizó mediante racimos y estratificación en 3 estratos diferentes, utilizando un porcentaje de confianza del 95% y un error máximo del 5%. Para cada estrato municipal, el error máximo era del 10%.

### Variables de estudio

Las variables analizadas fueron: socioeconómica: edad (14/15, 16/17, 18/19 años); identidad de género, en la que cisgénero se clasificaba como una identidad de género correspondiente a la asignada al nacer, clasificada como cisgénero o no cisgénero; raza/color de piel (blanco, negro, amarillo, marrón, autóctono); está en una relación (no, sí); tipo de colegio (público, privado); clasificación socioeconómica (A, B, C, D/E) - para esta variable, utilizamos el criterio de la Asociación Brasileña de Empresas de Investigación^9^; religión (ninguna, católica, evangélica, espiritista, de raíz africana, otra); tiene un trabajo/prácticas (no, sí); el estado de los padres (viven juntos, viven separados, uno de ellos ya ha fallecido - o ambos); Conductual: ¿Alguna vez has tenido relaciones sexuales (no, sí); uso de preservativos en la primera (no, sí); uso de preservativos en la última relación sexual (no, sí); experimentación con drogas (no, sí); consumo actual de drogas (no, sí); experimentación con cigarrillos (no, sí); uso actual de cigarrillos (no, sí); experimentación con bebidas alcohólicas (no, sí); el uso actual de bebidas alcohólicas (no, sí); Violencia entre iguales: víctima de acoso escolar (no, sí); acosado (no, sí); víctima de ciberacoso (no, sí); ciberacoso (no, sí); la frecuencia con la que participaba en peleas físicas (nunca se involucró, lo hizo una o más veces); Tipos de acoso sufrido: golpes/golpes/patadas/atrapados en lugares cerrados/empujados (no, sí); Se burlaban de él por raza, nacionalidad o color de piel (no, sí); Se burlaban de él por la religión (no, sí); Era objeto de burlas con bromas o comentarios de naturaleza sexual o gestos obscenos (no, sí); Fue excluido de actividades a propósito o completamente ignorado (no, sí); Se burlaban de él por la apariencia de su cuerpo o su rostro (no, sí); sufrió otra forma de acoso (no, sí); Motivación para el acoso: color/raza (no, sí); religión (no, sí); apariencia del rostro (no, sí); apariencia corporal (no, sí); orientación sexual (no, sí); región de origen (no, sí); Otros (no, sí).

### Instrumentos utilizados para recopilar información

La recogida de datos se realizó mediante cuestionarios electrónicos autocompletados en tabletas utilizando el sistema RedCAP (*Research Electronic Data Capture*), compuesto por bloques de caracterización socioeconómica y conductual. Además, se rastrearon las experiencias de violencia entre iguales y sus motivaciones utilizando un instrumento basado en el *Adverse Childhood Experiences International Questionnaire* (ACE-IQ), adaptado y validado para su uso en Brasil^10^.

### Recopilación de datos

La recopilación de datos fue realizada por un equipo responsable, formado y supervisado para garantizar la calidad e integridad de la información. Antes del inicio de la investigación, se solicitó y obtuvo autorización de la *Secretaria de Estado da Educação* (SEDU) para llevar a cabo el estudio en las escuelas públicas seleccionadas. Después, se estableció un contacto previo con instituciones públicas, así como con escuelas privadas, para presentar la propuesta en un seminario celebrado en marzo de 2023, donde participaron aproximadamente el 80% de las escuelas seleccionadas para la investigación. En esta ocasión, se presentaron los objetivos y procedimientos de la investigación y se entregaron los Formularios de Consentimiento Informado (FCI) a los representantes de las instituciones. Para las escuelas ausentes, el equipo realizó visitas presenciales, asegurando el cumplimiento del 100% de las unidades seleccionadas. Posteriormente, se estableció un nuevo contacto para la recogida en las escuelas del FCI y la programación de la recogida de datos, que se realizaba en el aula y/o auditorio, según la disponibilidad del centro. Es importante destacar que, en el momento de la recogida de datos, el equipo distribuyó una tableta a cada estudiante participante y proporcionó orientación sobre el uso del material. El tiempo medio para rellenar el cuestionario fue de unos cincuenta minutos.

### Tratamiento y análisis de datos

Se realizaron análisis estadísticos descriptivos utilizando frecuencias brutas y relativas e intervalos de confianza del 95% (IC 95%). Todos los análisis se realizaron utilizando el programa estadístico Stata 17.0.

### Aspectos éticos

Este estudio fue aprobado por el Comité de Ética en Investigación de la Universidad Federal de Espírito Santo, de conformidad con la Resolución 466/2012 del Consejo Nacional de Salud. Los padres y/o tutores de los adolescentes participantes firmaron el Formulario de Consentimiento Informado (FCI) y los adolescentes, el Término de Asentimiento Libre y Esclarecido (TALE). Cabe señalar que esta investigación fue financiada por la *Fundação de Amparo à Pesquisa do Espírito Santo* (FAPES) bajo el número de proceso CAAE 65545622.5.0000.5060.

## Resultados

Las características socioeconómicas de los estudiantes autodeclarados LGBT+ en la RMGV (Tabla 1) mostraron que la mayoría se declaraba negra (morena - 38,9%; negra - 18,5%). Predominaban los adolescentes de 16 a 17 años (65,5%), que no estaban en una relación (69,0%), cisgénero (81,0%) y asistían a escuelas públicas (67,2%).

Se observa que el 44,7% pertenecía a las clases B y el 45,3% no tenía religión. Finalmente, el 76,7% no trabajaba y, en el estado de sus padres, el 47,1% vivía por separado.

**Tabla 1:** Características socioeconómicas de los estudiantes LGBT+ autodeclarados. Vitória, ES, Brasil, 2023

Variable	N*	%^†^	IC95%^‡^
Grupo de edad (años)			
14 a 15	204	21,5	19,0-24,2
16 a 17	621	65,5	62,4-68,5
18 a 19	123	13,0	11,0-15,2
Identidad de género			
Cisgénero	769	81,0	78,4-83,4
No cisgénero	180	19,0	16,6-21,6
Raza/Color			
Blanca (o)	380	40,0	37,0-43,2
Negra (o)	175	18,5	16,1-21,0
Amarilla (o)	15	1,6	0,9-2,0
Mestiza (o)	369	38,9	35,8-42,0
Indígena	10	1,0	0,5-2,0
¿Tiene pareja?			
No	655	69,0	66,0-71,8
Sí	294	31,0	28,1-33,9
Escuela pública o privada			
Pública	638	67,2	64,1-70,1
Privada	311	32,8	29,9-34,6
Clasificación socioeconómica			
A	154	16,3	14,0-18,7
B	424	44,7	41,6-47,9
C	317	33,5	30,5-36,5
D/E	53	5,5	4,2-7,2
Religión			
Ninguna	429	45,3	42,0-48,4
Católica	167	17,6	15,3-20,1
Evangélica	215	22,6	20,1-25,4
Espiritista	54	5,7	4,4-7,4
Raiz africana	48	5,0	3,8-6,6
Otra	36	3,8	2,7-5,2
Tiene trabajo/pasantía			
No	728	76,7	74,0-79,3
Sí	221	23,3	20,7-26,0
Situación de los padres			
Viven juntos	424	45,1	41,9-48,3
Viven separados	443	47,1	43,9-50,3
Uno de ellos ya falleció (o los dos)	73	7,8	6,2-9,6

*N = Frecuencia absoluta; ^†^% = Prevalencia; ^‡^IC95% = Intervalo de confianza del 95%

Las características conductuales de los estudiantes autodeclarados LGBT+ en la RMGV se presentan en la Tabla 2. En cuanto a la experiencia sexual, la mayoría (56,6%) nunca había tenido relaciones sexuales, mientras que el 43,4% afirmó que ya había tenido una. El uso de preservativos durante la primera relación sexual fue reportado por aproximadamente el 51,0% de los adolescentes (IC 95%: 45,9-55,5); sin embargo, en la última relación sexual, aproximadamente el 40,0% reportó haberla usado (IC 95%: 44,4-54,1). En cuanto al consumo de drogas a lo largo de la vida, el 33,6% ya las ha consumido y, actualmente, el 15,1% las consume. Se ha verificado que el 31,8% ya ha fumado y actualmente esta frecuencia es del 9,1%. En cuanto al consumo de bebidas alcohólicas a lo largo de la vida, el 77,2% ya las ha consumido y el 47,2% las utiliza actualmente.

**Tabla 2:** Características conductuales de los estudiantes autodeclarados LGBT+. Vitória, ES, Brasil, 2023

Variable	N*	%^†^	IC95%^‡^
¿Alguna vez has tenido relaciones sexuales?			
No	537	56,6	53,4-59,7
Sí	412	43,4	40,3-46,6
Uso de preservativo durante la primera relación sexual			
No	203	49,3	44,4-54,1
Sí	209	50,7	45,9-55,5
Uso de preservativo en la última relación sexual			
No	246	59,7	54,9-64,3
Sí	166	40,3	35,7-45,1
Ha probado drogas			
No	630	66,4	63,3-69,3
Sí	319	33,6	30,7-36,7
Consumo de drogas hoy en día			
No	805	84,8	82,4-87,0
Sí	144	15,2	13,0-17,6
Ha probado cigarrillos			
No	647	68,2	65,1-71,0
Sí	302	31,8	29,0-34,8
Uso actual de cigarrillos			
No	863	90,9	89,0-92,6
Sí	86	9,1	7,3-11,0
Ha probado bebidas alcohólicas			
No	216	22,8	20,2-25,6
Sí	731	77,2	74,4-79,7
Consumo actual de alcohol			
No	500	52,8	49,6-56,0
Sí	447	47,2	44,0-50,4

*N = Frecuencia absoluta; ^†^% = Prevalencia; ^‡^IC95% = Intervalo de confianza del 95%

En cuanto a la prevalencia de la violencia entre iguales (Tabla 3), el 67,8% de los estudiantes LGBT+ declaró haber sido víctima de acoso escolar y el 11,5% reportó haber sufrido ciberacoso. En cuanto al acoso escolar, el 15,2% de los participantes admitió haber cometido actos de esta naturaleza, mientras que el 7,0% reportó la práctica del ciberacoso. Además, alrededor del 38,0% reportó haber participado en peleas físicas al menos una vez.

**Tabla 3:** Prevalencia del acoso y el ciberacoso entre estudiantes que se declararon LGBT+. Vitória, ES, Brasil, 2023

Variable	N*	%^†^	IC95%^‡^
Víctima de acoso escolar			
No	306	32,2	29,3-35,3
Sí	643	67,8	64,7-70,7
Practicó acoso escolar			
No	803	84,8	82,4-86,9
Sí	144	15,2	13,1-17,6
Víctima de ciberacoso			
No	838	88,5	86,3-90,4
Sí	109	11,5	9,6-13,7
Practicó ciberacoso			
No	881	93,0	91,2-94,5
Sí	66	7,0	5,5-8,8
Frecuencia con la que se ha visto involucrado en peleas físicas			
Nunca se involucró	569	62,3	59,1-65,3
Se involucró una o más veces	345	37,7	34,7-40,9

*N = Frecuencia absoluta; ^†^% = Prevalencia; ^‡^IC95% = Intervalo de confianza del 95%

En cuanto a los tipos de acoso escolar (Tabla 4), se observa que el 8,0% de los participantes informó haber sufrido agresión física, como ser pateados, empujados o encerrados en espacios cerrados. Aproximadamente el 19,0% mencionó insultos relacionados con raza, nacionalidad o color de piel. El 5,5% reportó comentarios de carácter religioso y el 17,3% señaló chistes o gestos obscenos de índole sexual. Además, el 29,2% reportó haber sido excluido de actividades o completamente ignorado, mientras que el 44,3% sufrió insultos relacionados con la apariencia física y otras formas de acoso escolar fue identificado por un 12,9%.

**Tabla 4:** Tipos de acoso escolar practicados contra os estudiantes que se declararon LGBT+. Vitória, ES, Brasil, 2023

Variable	N*	%^†^	IC95%^‡^
Golpeaban/Empujaban/Pateaban/Encerraban en lugares cerrados/Empujaban			
No	873	92,0	90,0-93,5
Sí	76	8,0	6,4-10,0
Era objeto de burlas (se mofaban de él/ella) por su raza, nacionalidad o color de piel.			
No	770	81,1	78,5-83,5
Sí	179	18,9	16,5-21,5
Era objeto de burlas (se mofaban de él/ella) por su religión			
No	897	94,5	92,8-95,8
Sí	52	5,5	4,2-7,1
Era objeto de burlas (se mofaban de él/ella) mediante bromas o comentarios de carácter sexual o gestos obscenos.			
No	785	82,7	80,1-85,0
Sí	164	17,3	15,0-19,9
Fue excluido de actividades a propósito o completamente ignorado			
No	672	70,8	67,8-73,6
Sí	277	29,2	26,4-32,2
Se burló de él (se burló) por la apariencia de su cuerpo o rostro			
No	529	55,7	52,6-58,9
Sí	420	44,3	41,1-47,4
Sufría otra forma de acoso escolar			
No	827	87,1	84,8-89,1
Sí	122	12,9	10,9-15,1

*N = Frecuencia absoluta; ^†^% = Prevalencia; ^‡^IC95% = Intervalo de confianza del 95%

El análisis de las motivaciones para el acoso escolar entre adolescentes LGBT+ (Tabla 5) reveló que el 5,0% de los participantes mencionó el color o la raza, mientras que la religión se reportó en un 4,2%. En cuanto a la apariencia del rostro, el 9,1% de los adolescentes reportó haber sido acosado por esta razón y el 10,4% indicó la apariencia del cuerpo como un factor motivador. Cabe destacar que la orientación sexual fue la que tuvo la mayor prevalencia entre las razones presentadas, siendo mencionada por el 13,0% de los participantes.

**Tabla 5:** Motivaciones para la ocurrencia de acoso escolar contra estudiantes autodeclarados LGBT+. Vitória, ES, Brasil, 2023

Variable	N*	%^†^	IC95%^‡^
Color/Raza			
No	902	95,0	93,4-96,2
Sí	47	5,0	3,7-6,5
Religión			
No	909	95,8	94,3-96,8
Sí	40	4,2	3,1-5,6
Apariencia facial			
No	863	90,9	88,9-92,6
Sí	86	9,1	7,4-11,0
Apariencia corporal			
No	850	89,6	87,4-91,3
Sí	99	10,4	8,6-12,5
Orientación sexual			
No	826	87,0	84,7-89,0
Sí	123	13,0	10,9-15,2
Región de origen			
No	922	97,1	95,8-98,0
Sí	27	2,8	1,9-4,1
Otros			
No	862	90,8	88,8-92,5
Sí	87	9,2	7,4-11,1

*N = Frecuencia absoluta; ^†^% = Prevalencia; ^‡^IC95% = Intervalo de confianza del 95%

## Discusión

Los datos socioeconómicos presentados revelan aspectos importantes sobre el perfil de los escolares LGBT+ autodeclarados en Espírito Santo. Se observa que el 65,5% pertenecía al grupo de edad de 16 y 17 años, predominantemente cisgénero (81,0%), lo que refleja la tendencia mayoritaria en muchas sociedades, aunque una parte significativa (19,0%) se identificaba como no cisgénero, lo que pone de manifiesto la diversidad de identidades de género dentro de este grupo^11^. Además, el 69,0% de los participantes no estaban en una relación, lo que puede indicar un patrón de relaciones o una etapa de la vida en la que la mayoría aún no ha mantenido relaciones formales, algo común en los grupos de edad más jóvenes^12^.

En cuanto a religión, el 45,3% de los adolescentes declaró no tener religión. Muchos adolescentes LGBT+ experimentan un conflicto entre sus identidades sexuales y religiosas, lo que a menudo resulta en sentimientos de desconexión con sus comunidades religiosas^11^. Relatan experiencias de conexiones iniciales con su fe, pero más tarde empiezan a cuestionar sus creencias debido a su orientación sexual^13^.

Al comparar los datos obtenidos en el estudio con otros estudios en Brasil, se observa que la prevalencia de iniciación sexual en el presente estudio entre los adolescentes de la muestra es considerablemente mayor (43,4%). Un estudio realizado en Piauí, con adolescentes sin orientación sexual, indicó una prevalencia del 24,2%^14^ y, en Curitiba, también entre la población general de adolescentes, la prevalencia fue del 22,9%^15^. Estos datos sugieren que la iniciación sexual es un fenómeno presente en la adolescencia LGBT+, con la orientación sexual como un factor que puede influir en la edad de inicio, reforzando la importancia de los programas de educación sexual para este público. 

En este estudio, aproximadamente el 51,0% de los participantes informó haber usado preservativos durante su primera relación sexual, un hallazgo superior al identificado en un estudio con adolescentes de la población general (24,7%)^16^. Por otro lado, en la última relación sexual, la prevalencia de consumo fue del 40,3%, un porcentaje inferior al encontrado en otro estudio realizado con adolescentes de la población general (68,8%)^17^. Estos hallazgos sugieren que los adolescentes LGBT+ tienden a adoptar una mayor protección al inicio de su vida sexual, motivados principalmente por la prevención de ITS, la desconfianza o la imposición hacia la pareja. Sin embargo, la reducción en la adhesión al preservativo en relaciones posteriores puede estar relacionada con la confianza en la pareja, preferencias personales, olvido o falta de disponibilidad del método^18^.

En vista de lo anterior, es importante llamar la atención sobre el trabajo de educación para la salud que debe realizarse en el entorno escolar por parte de los profesionales sanitarios, precisamente para que la adhesión a los métodos anticonceptivos se extienda más allá del primer coito sexual, porque, a través de esta acción, se mejora la comprensión de la salud sexual por parte de los adolescentes, lo que conduce a buenas actitudes y comportamientos en relación con las ITS^19^.

En cuanto a las drogas, el 33,6% declaró haberlos probado y actualmente el 15,1% los consume. Comparando estos hallazgos, hay estudios que indican que el 20,9% de los adolescentes, sin distinción de orientación sexual, reportan el consumo de drogas ilícitas a lo largo de su vida, y una prevalencia del 15,8% del consumo actual de drogas, como la marihuana, en la adolescencia^1^ ^,^ ^20^. Los altos niveles encontrados en la población LGBT+ se analizan en la literatura a la luz del rechazo y la discriminación que sufre esta población, estrechamente vinculados al aumento de problemas de salud mental, reconocidos como determinantes importantes del consumo de sustancias entre los adolescentes LGBT+^21^. Además, las experiencias recurrentes de acoso escolar y conductas opresivas intensifican los sentimientos de aislamiento y angustia, creando un entorno propicio para el uso inapropiado de sustancias como forma de afrontar o aliviar temporalmente las adversidades^22^.

Se ha verificado que el 31,8% ya ha fumado y, actualmente, esta frecuencia es del 9,1%. Un estudio indica que el 22,58% de los adolescentes de la población general declaró haber probado tabaco, mientras que el consumo actual es aproximadamente del 6,8%^23^, prevalencias menores que las encontradas en el presente estudio, lo que muestra una mayor vulnerabilidad de los adolescentes LGBT+ autodeclarados al tabaquismo. Las experiencias de discriminación por orientación sexual e identidad de género aumentan significativamente la probabilidad de consumo de tabaco entre los jóvenes LGBT+^24^.

Los datos sobre el consumo de alcohol en nuestra muestra revelan que aproximadamente el 77,0% de los participantes reportaron experimentación con alcohol al menos una vez en su vida, una alta prevalencia al comparar el resultado con otros hallazgos, que encontraron el 52,9% de la experimentación por adolescentes brasileños de la población general^25^. El consumo actual de alcohol fue mencionado por un 47,2%, lo que también es una prevalencia alta en comparación con el consumo total de adolescentes en la población general (25,2%)^26^. Se destaca una preocupante realidad de nuestra población estudiada, que sugiere que factores sociales, como la experiencia de estigmas y desafíos relacionados con la orientación sexual, pueden influir en este comportamiento^27^.

En el entorno escolar, el acoso escolar se configura como una expresión de esta violencia estructural, revelando una realidad alarmante. Los datos indican que la LGBTfobia es la tercera causa principal de acoso escolar, con un 73,0% de estudiantes LGBT+ que reportan agresión verbal y un 36,0% que reportan agresión física^28^. Estos hallazgos están corroborados por el presente estudio, en el que el 67,8% (IC 95%: 64,7-70,7) de los adolescentes LGBT+ reportó haber sido víctima de acoso escolar y el 37,8% (IC 95%: 34,7-40,9) admitió haber participado en peleas físicas.

La violencia contra los adolescentes LGBT+ en las escuelas representa un tema relevante, especialmente si se consideran las múltiples formas de agresión que enfrentan estas personas y el impacto negativo de estas experiencias en su salud mental y bienestar social. Los estudios indican un aumento de estos episodios, reflejando la discriminación estructural que aún impregna la sociedad brasileña^29^ ^-^ ^30^.

Observamos una alta prevalencia de ciberacoso entre los adolescentes LGBT+ (11,5%), un valor significativo en comparación con otras realidades adolescentes internacionales y nacionales. Un estudio realizado en Yixing, China, informó de una tasa general de victimización por ciberacoso de solo el 2,9% entre adolescentes, mostrando una diferencia notable respecto a la población estudiada^31^. Un análisis exhaustivo de los datos de la *Pesquisa Nacional de Saúde do Estudante* de 2019 (PeNSE) reveló una prevalencia del 13,2% entre escolares brasileños de entre 13 y 17 años^32^. Estos datos sugieren que, aunque el ciberacoso es un problema global, su magnitud puede variar significativamente según el contexto sociocultural.

En cuanto al acoso escolar, el 15,2% de los participantes admitió haber cometido actos de esta naturaleza, mientras que el 7,0% reportó la práctica del ciberacoso. En este contexto, factores como la ira, los problemas familiares y la reproducción de la violencia sufrida emergen como pilares de conflicto entre iguales^33^, especialmente entre adolescentes LGBT+, que, a pesar de ser a menudo víctimas de esta violencia, también pueden, en ciertos casos, reproducirla como respuesta a sus propias experiencias de marginación y sufrimiento^34^.

Además, el 8,0% de los participantes reportó haber sufrido agresión física, como patadas, empujones o encerrados en casa. Estos comportamientos no solo son indicativos de violencia escolar, sino que también pueden reflejar un entorno educativo en el que la resolución pacífica de conflictos no se fomenta adecuadamente. La práctica de peleas físicas puede provocar traumas tanto físicos como emocionales, con consecuencias duraderas para el desarrollo académico de los adolescentes^35^.

Aproximadamente el 19,0% de los adolescentes mencionó insultos relacionados con la raza, la nacionalidad o el color de piel. El racismo y la xenofobia son formas de violencia psicológica que tienen un impacto directo en la construcción de la identidad individual y pueden contribuir al aumento del aislamiento social y la exclusión escolar^36^. Además, se destaca que los estereotipos negativos en las escuelas contribuyen significativamente al riesgo de acoso escolar entre minorías raciales y étnicas^37^.

El 5,5% reportó comentarios de carácter religioso y el 17,3% señaló chistes o gestos obscenos de índole sexual. Los comentarios religiosos, además de ser una forma de discriminación, pueden agravar los conflictos de identidad, especialmente en una sociedad plural como la nuestra^38^. Los gestos o comentarios sexuales, en cambio, son formas sutiles de violencia sexual, a menudo minimizadas, pero con un profundo impacto en el desarrollo emocional y psicológico de los jóvenes. Estos juegos o gestos, aunque no sean físicamente invasivos, pueden causar daños duraderos a la autoestima y la salud mental, y es esencial que se identifiquen y aborden en entornos escolares^39^.

Los adolescentes LGBT+ a menudo sufren acoso escolar relacionado con su aspecto, como críticas a la forma corporal y los rasgos faciales, influenciadas por los estándares sociales de belleza. En este contexto, los hallazgos revelan una prevalencia significativa (44,3%) de adolescentes que reportan ser objeto de burlas relacionadas con la apariencia física. Este tipo de acoso escolar puede tener impactos duraderos en la autoestima y el bienestar de los jóvenes, reforzando la necesidad de acciones educativas y de apoyo para combatir el estigma y promover la aceptación de la diversidad en las escuelas y la sociedad^40^.

Del mismo modo, la prevalencia de la exclusión social, que se dice es una motivación para ser excluidos de actividades a propósito o completamente ignorados (29,2%), llama nuestra atención. Las actitudes sociales entre iguales pueden llevar a la exclusión de personas LGBT+, con adolescentes que perciben que es más aceptable excluir a iguales gays o lesbianas en comparación con heterosexuales^41^. Las consecuencias de la exclusión social son graves, con adolescentes LGBT+ que experimentan tasas más altas de ansiedad, depresión y pensamientos suicidas^42^.

Aún analizando las motivaciones que llevaron a los adolescentes LGBT+ a sufrir acoso, se observa que el 13,0% de los participantes reportó haber sufrido violencia por haber declarado su orientación sexual. Los investigadores analizaron una muestra de 815 adolescentes e identificaron que quienes se declaraban homosexuales o bisexuales presentaban relaciones inversas significativas entre la victimización homófoba y la calidad de vida relacionada con la salud, lo que evidenciaba los efectos perjudiciales de este tipo de acoso^43^. El acoso homófobo es especialmente perjudicial, lo que resulta en una reducción significativa de la calidad de vida y afecta negativamente el estado de ánimo, las emociones y la aceptación social de los adolescentes afectados^42^.

Los resultados de este estudio ponen de manifiesto la importancia de intervenciones específicas por parte de profesionales de la salud y la educación, que son esenciales para prevenir la violencia y promover entornos inclusivos para los adolescentes LGBT+. La integración entre estos sectores puede potenciar el impacto de los programas educativos y de sensibilización, favoreciendo la construcción de un entorno escolar acogedor y seguro. Acciones como la formación de profesores y gestores escolares, combinadas con apoyo psicológico a los estudiantes, son esenciales para reducir las tasas de acoso y discriminación.

Es importante destacar las limitaciones inherentes al diseño del estudio, con posibles sesgos de memoria e información. Sin embargo, la relevancia de la investigación radica no solo en el uso de una muestra exhaustiva, incluyendo escuelas públicas y privadas, sino también en el uso de cuestionarios autoadministrados que permiten una mayor confidencialidad y precisión en las respuestas.

Los hallazgos proporcionan un apoyo valioso para el desarrollo de políticas públicas e intervenciones destinadas a promover la equidad y la salud entre la población adolescente LGBT+, además de animar a los profesionales sanitarios, especialmente a la enfermería, a utilizar esta información para desarrollar estrategias acogedoras para este grupo.

## Conclusión

Este estudio mostró que el perfil socioeconómico de los adolescentes LGBT+ en el RMGV está compuesto principalmente por personas negras de 16 a 17 años, estudiantes de escuelas públicas y pertenecientes a la clase socioeconómica B. Además, se observa la presencia de conductas de riesgo relacionadas con el uso inapropiado de preservativos y el consumo de sustancias psicoactivas. Reportan experiencias de violencia, con alta prevalencia de acoso escolar, ciberacoso y exclusión social, la mayoría motivadas por la declaración de su orientación sexual.

Es innegable que la adolescencia es una fase de descubrimiento y vulnerabilidad, y para los adolescentes LGBT+, estos desafíos se ven amplificados por estigmas y barreras estructurales. En vista de ello, es esencial que educadores, profesionales sanitarios y gestores públicos actúen de manera integrada, promoviendo entornos más inclusivos y seguros, en los que la diversidad sea respetada y celebrada. Garantizar el bienestar de los adolescentes LGBT+ no es solo un problema de salud pública, sino un compromiso con la equidad y los derechos humanos.

La implementación de políticas públicas basadas en estos datos sigue enfrentando desafíos significativos, como la resistencia sociocultural a la inclusión de cuestiones de diversidad sexual y de género en el entorno escolar y en los servicios de salud. Superar estas barreras requiere acciones intersectoriales coordinadas, formación continua de profesionales y participación comunitaria.

## Data Availability

Los conjuntos de datos relacionados con este artículo estarán disponibles previa solicitud al autor correspondiente.
